# Can a simple fall cause a rotator cuff tear? Literature review and biomechanical considerations

**DOI:** 10.1007/s00264-021-05012-6

**Published:** 2021-03-27

**Authors:** Richard W. Nyffeler, Nicholas Schenk, Philipp Bissig

**Affiliations:** 1 Orthopädie Sonnenhof, Salvisbergstrasse 4, 3006 Bern, Switzerland; 2 Stiftung Lindenhof, Campus SLB, Swiss Institute for Translational and Entrepreneurial Medicine, Freiburgstrasse 3, 3010 Bern, Switzerland; 3 Praxis Integri, Hirschengraben 7, 3011 Bern, Switzerland

**Keywords:** Shoulder, Trauma, Injury, Fall, Rotator cuff tear

## Abstract

**Purpose:**

A simple fall on the shoulder is often referred to as minor trauma that cannot cause a tendon tear but at best reveal a pre-existing rotator cuff pathology. We wanted to know whether this statement was true. The purpose of our study was therefore to summarize the causes of acute rotator cuff tears reported in the literature and provide a biomechanical explanation for tendon tears diagnosed after a fall.

**Method:**

We searched PubMed and included studies reporting rotator cuff tears occurring due to a trauma. The number of cases, the tendons involved, the age of the patients, and the nature of trauma were summarized. In addition, we noted any information provided by the authors on the pathogenesis of acute tendon ruptures.

**Results:**

Sixty-seven articles with a total of 4061 traumatic rotator cuff tears met the inclusion criteria. A simple fall was the most common cause (725 cases) and the supraspinatus tendon was most frequently affected. The postulated pathomechanism is a sudden stretch of the tendon-muscle unit while contracting (eccentric loading).

**Conclusion:**

A simple fall can cause an acute rotator cuff tear and fall-related tears are not restricted to young individuals. They can affect patients of any age. The stresses occurring within the rotator cuff during an attempt to cushion a fall may locally exceed the tensile strength of the tendon fibers and cause a partial or full-thickness tear.

## Introduction

Rotator cuff tear (RCT) is a common cause of shoulder pain and dysfunction in patients older than 50 years of age. The prevalence increases with age [1–4], the aetiology, however, is still under debate. Many authors hold the opinion that the majority of tendon tears are the result of age-related degenerative changes [5]. Accordingly, research has focused on this area and numerous intrinsic and extrinsic factors for tendon degeneration have been postulated in the last decades. Accident-related research has not been advanced to the same extent, although more than 80 years ago, some eminent scientists reported that, in addition to age-related tissue changes, a definite injury is required for a full-thickness RCT [6–8].

**Fig. 1:**
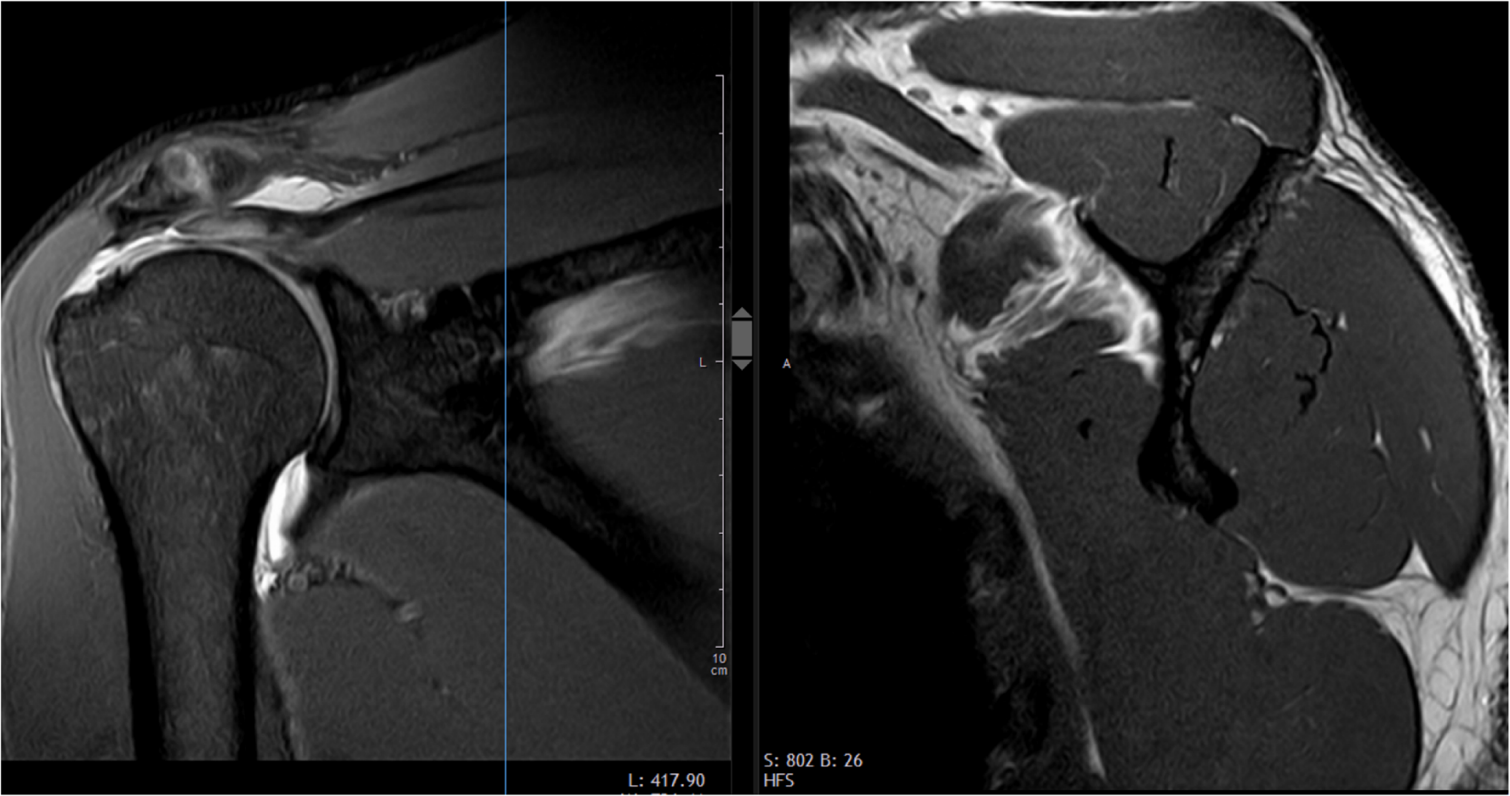
Coronal and sagittal MR images of the right shoulder of a 37-year-old man showing a full-thickness supraspinatus tendon tear. The examination was made seven weeks after a fall on the shoulder following a header duel during a football match. Despite the young age, immediate pain, initial pseudoparesis, and normal muscle trophics, the insurance expert claimed that the accident had only caused a contusion and that the tendon was already torn before the event

The assessment of whether a tendon lesion is traumatic or degenerative in nature is not only important to determine the type of treatment required and expected outcome [9–15], but also in applying the appropriate insurance cover. In many countries, the benefits of accident insurances are more comprehensive than those of health insurances. Due to the high prevalence of RCTs in the elderly population, as well as the major economic burden they pose, accident insurance companies are very critical of accident-related tendon tears. Certain experts [16–18] have maintained for many years that a fall onto the outstretched arm or the shoulder cannot cause an RCT, but will only result in a contusion. They argued that the deltoid muscle covers and protects the rotator cuff, that the scapula can deflect and absorb the energy, and that the tensile strength of the rotator cuff tendons is three times higher than the maximum force the muscles can generate. They also purport that most RCTs, which are diagnosed on MRI after a fall, are pre-existing due to degenerative changes or overuse (Fig. 1). Courts often uphold insurance experts, even if the patients had not experienced any previous shoulder complaints and had never consulted their family doctor for shoulder problems prior to the accident. The purpose of the present study was therefore to review the literature on acute and traumatic RCTs, summarize the nature of trauma, and provide a biomechanical explanation for the tendon tears caused by falls.

## Materials and methods

We conducted a literature search of PubMed up to September 30, 2020, using the terms “acute rotator cuff tear,” “traumatic rotator cuff tear,” and “fall AND rotator cuff tear.” This yielded 450 results after exclusion of duplicates. The title and abstract of each result were examined. Animal studies, cadaver studies, technical reports, case reports, review articles, and studies without acute or traumatic tears were excluded. The full texts of eligible articles were screened, and the following information was extracted: authors, year of publication, study design (retrospective versus prospective), number and age of patients included, number or percentage of traumatic tendon tears, affected tendons and causes.

## Results

Sixty-seven articles with a total of 10,796 shoulders met the inclusion criteria. They are listed chronologically in Table 1. Most studies were retrospective. Eighteen studies included only traumatic tears. The others included also patients with non-traumatic tears, other pathologies, or healthy individuals in a control group. Overall, 4061 RCTs were attributed to trauma. The following causes were reported in descending order of frequency: fall (725), shoulder dislocation (577), violent pull or sudden traction injury (296), sports injury (145), blow, direct trauma or impact to the shoulder (103), vehicle accident (97), hyperextension, forced abduction and external rotation or grabbing a rail to prevent falling (88), and lifting heavy objects or catching a falling object (29). The mechanism of injury in traffic accidents, sports injuries, and the causes of shoulder dislocations were not always specified. It can be assumed that a considerable number of them were the result of a fall [56]. In 2001 cases, the nature of trauma was not clear or not reported. The supraspinatus was by far the most frequently affected tendon. Bigger tears also involved the infraspinatus and or subscapularis tendon. Isolated tears of the subscapularis tendon were also reported. The age of patients with fall-related RCTs ranged from 15 to 89 years [66].

**Table 1:** Summary of included studies with traumatic rotator cuff tears, sorted by year of publication. The number of patients, their age, and the nature of trauma are listed. Abbreviations: *ABER*, forced abduction external rotation movement, *n.r.*, not reported

Authors and year	Shoulders included	Age entire study population		Traumatic tears		Nature of trauma								
	*n*	Mean	Range	*n*	(%)	Fall	Shoulder dislocation	Violent pull, sudden traction	Sports injury	Blow, impact to shoulder	Vehicle accident	Hyperext., ABER, grabbing a rail	Lifting heavy objects, catching a falling object	Others, not clear, not reported
Lindblom [8], 1939	50	n.r	(20–80)	45	(90)	38	-	-	-	-	-	-	-	7
Bassett et al. [9], 1983	37	56	(19–74)	36	(97)	22	-	-	4	-	1	3	6	-
Gerber et al. [19], 1991	16	50	(33–60)	16	(100)	-	1	-	-	-	-	9	-	6
Bigliani et al. [20], 1992	23	58	(39–71)	11	(48)	-	-	-	6	-	-	-	-	5
Bigliani et al. [21], 1992	61	62	(40–77)	30	(49)	-	4	-	-	-	-	-	-	26
Bokor et al. [22], 1993	53	62	(45–83)	40	(75)	9	12	8	-	11	-	-	-	-
Blevins et al. [23], 1996	10	26	(24–36)	8	(80)	-	-	-	-	8	-	-	-	-
Iannotti et al. [24], 1996	40	55	(39–71)	27	(68)	-	-	-	-	-	-	-	-	27
Berbig et al. [2], 1999	167	55	(16–92)	53	(32)	-	53	-	-	-	-	-	-	-
Habernek et al. [25], 1999	39	48	(30–69)	19	(49)	-	-	-	-	-	-	-	-	19
Hawkins et al. [26], 1999	19	33	(23–40)	16	(84)	7	5	-	2	-	1	-	1	-
Hersch et al. [27], 2000	22	56	(29–80)	15	(68)	13	-	-	-	-	2	-	-	-
Teefey et al. [28], 2000	44	n.r.	(31–83)	24	(55)	-	-	-	-	-	-	-	-	24
Foulk et al. [29], 2002	51	n.r.	n.r.	43	(84)	20	1	-	-	3	-	8	-	11
Braune et al. [11], 2003	46	45	(15–68)	20	(43)	-	-	-	-	-	-	-	-	20
Goldberg et al. [30], 2003	6	27	(23–29)	6	(100)	2	-	-	-	-	-	-	-	4
Kim et al. [31], 2003	76	56	(42–75)	42	(55)	-	-	-	-	-	-	-	-	42
Mansat et al. [32], 2003	23	56	(34–69)	18	(78)	3	4	-	-	-	-	10	-	1
Sperling et al. [33], 2004	25	45	(30–50)	24	(96)	-	-	-	-	-	-	-	-	24
Kreuz et al. [34], 2005	34	51	(27–66)	34	(100)	-	-	-	-	13	2	19	-	-
McCabe et al. [35], 2005	61	52	n.r.	39	(64)	-	-	-	-	-	-	-	-	39
Lähteenmäki et al. [15], 2006	26	53	(25–68)	26	(100)	-	-	-	-	-	-	-	-	26
Lungren et al. [36], 2006	60	53	n.r.	60	(100)	32	-	-	-	-	-	-	-	28
Ide et al. [37], 2007	20	62	(45–79)	20	(100)	12	-	-	-	-	1	7	-	-
Sørensen et al. [38], 2007	104	49	(19–75)	60	(58)	40	-	11	-	-	-	-	-	9
Zingg et al. [39], 2007	19	64	(54–79)	16	(84)	-	-	-	-	-	-	-	-	16
Frank et al. [40], 2008	25	57	(44–74)	16	(64)	4	-	-	-	-	1	-	-	11
Krishnan et al. [41], 2008	23	37	(21–39)	22	(96)	-	2	-	-	-	-	-	-	22
Namdari et al. [42], 2008	30	57	(43–73)	30	(100)	-	7	-	-	-	-	-	-	28
Saupe et al. [43], 2008	36	40	(15–80)	7	(19)	-	-	-	-	-	-	-	-	-
Auplish et al. [44], 2009	11	26	n.r.	11	(100)	-	-	-	11	-	-	-	-	-
Berhouet et al. [10], 2009	112	56	(35–65)	57	(51)	-	1	-	-	-	-	-	-	57
Tambe et al. [45], 2009	11	27	(19–31)	11	(100)	-	-	-	-	6	-	3	-	1
Bak et al. [46], 2010	52	51	(19–75)	29	(56)	-	-	-	-	-	-	-	-	29
Didden et al. [47], 2010	73	49	(36–55)	42	(58)	-	-	-	-	-	-	-	-	42
Melis et al. [48], 2010	1688	57	n.r.	669	(40)	-	-	-	-	-	-	-	-	669
Moosmayer et al. [49], 2010	103	60	(44–75)	40	(39)	-	-	-	-	-	-	-	-	40
Tanaka et al. [50], 2010	128	69	(42–83)	28	(22)	-	1	-	-	-	-	-	-	28
Bartl et al. [51], 2011	30	43	(15–64)	30	(100)	6	-	-	-	2	1	18	-	2
Bartl et al. [52], 2011	21	44	(18–61)	19	(90)	8	17	-	-	1	-	10	-	-
Björnsson et al. [53], 2011	42	59	(38–79)	42	(100)	-	8	-	-	-	-	-	-	25
Hantes et al. [13], 2011	35	55	(28–70)	35	(100)	23	-	-	-	-	4	-	-	-
Meyer et al. [54], 2011	31	59	(45–75)	1	(3)	1	-	-	-	-	-	-	-	-
Petersen et al. [55], 2011	36	57	(21–74)	36	(100)	-	443	-	-	-	-	-	-	36
Robinson et al. [56], 2012	3633	48	(13–104)	443	(12)	-	-	-	-	-	-	-	-	-
Rousseau et al. [57], 2012	50	67	(46–80)	18	(36)	-	-	-	-	-	-	-	-	18
Kukkonen et al. [58], 2013	279	57	(26–80)	112	(40)	53	-	48	-	-	3	-	-	8
Lin et al. [59], 2013	53	37	(16–45)	32	(60)	8	-	-	12	-	-	-	10	2
Park et al. [60], 2013	36	62	(45–75)	2	(6)	-	-	-	-	-	-	-	-	2
Brogan et al. [61], 2014	280	33	(20–70)	23	(8)	5	-	-	-	-	13	-	-	5
Zbojniewicz et al. [62], 2014	205	n.r.	(10–18)	25	(12)	-	-	-	-	-	-	-	-	25
Aagaard et al. [63], 2015	259	51	(18–75)	60	(23)	48	-	-	-	-	-	-	-	12
Dilisio et al. [64], 2015	9	19	(13–25)	9	(100)	1	-	-	6	-	1	-	-	1
Dwyer et al. [65], 2015	344	n.r.	(24–90)	238	(69)	-	-	-	-	-	-	-	-	238
Tan et al. [66], 2016	1300	58	(15–89)	811	(62)	311	-	227	77	40	63	-	-	93
Abechain et al. [67], 2017	87	59	(40–76)	35	(40)	-	-	-	-	-	-	-	-	35
Callaghan et al. [68], 2017	20	44	(21–80)	12	(60)	5	3	2	-	2	-	1	2	-
Jeong et al. [14], 2017	72	61	n.r.	36	(50)	31	-	-	-	-	-	-	2	-
Simon et al. [69], 2017	12	55	(28–66)	6	(50)	-	-	-	-	-	-	-	-	6
Teratani [70], 2017	79	67	(48–85)	33	(42)	17	-	-	3	-	-	-	8	5
Walcott et al. [71], 2017	7	48	(33–71)	7	(100)	6	-	-	-	-	-	-	-	1
Aagaard et al. [72], 2018	184	n.r.	(18–75)	79	(43)	-	-	-	-	-	-	-	-	79
Azzam et al. [73], 2018	32	16	(13–18)	29	(91)	-	5	-	24	-	4	-	-	1
Haviv et al. [74], 2018	95	55	n.r.	37	(39)	-	-	-	-	17	-	-	-	15
Aagaard et al. [75], 2019	62	n.r.	(18–75)	62	(100)	-	10	-	-	-	-	-	-	62
Spross et al. [76], 2019	21	61	(30–83)	21	(100)	-	-	-	-	-	-	-	-	11
Ranebo et al. [77], 2020	58	60	(44–77)	58	(100)	-	-	-	-	-	-	-	-	58
Summary	10,796		(9–104)	4061	(37)	725	577	296	145	103	97	88	29	2001

Only few authors proposed a pathomechanism of traumatic supraspinatus tears. Lindblom [8] and Matsen [78] noted that falls on the outstretched hand or elbow are followed by a forced adduction of the arm in the scapulohumeral joint. Walcott et al. [71] hypothesized that a fall onto the abducted arm results in an axial load that forces the supraspinatus tendon into the lateral acromion, causing a transtendinous tear.

## Discussion

The primary finding of this literature review is that a substantial number of RCTs have been associated with a traumatic event. In the study by Tan et al. [66] including only patients with RCTs, the percentage of traumatic tendon tears was 62%, and in the study by Melis et al. [48], it was 40%. A history of trauma was also a risk factor for RCT in a large epidemiologic study on the prevalence of RCTs in the general population [4]. We found 26 articles that had associated RCTs with a fall (Table 1). Tan [66] reported 311, Kukkonen et al. [58] 53, Aagaard et al. [63] 48, and Lungren et al. [36] 32 RCTs after a fall. Foulk et al. [29] identified 51 professional athletes with RCTs and stated that the most common mechanism of injury was a fall. This statement is consistent with the findings of Mall et al. [79].

**Fig. 2:**
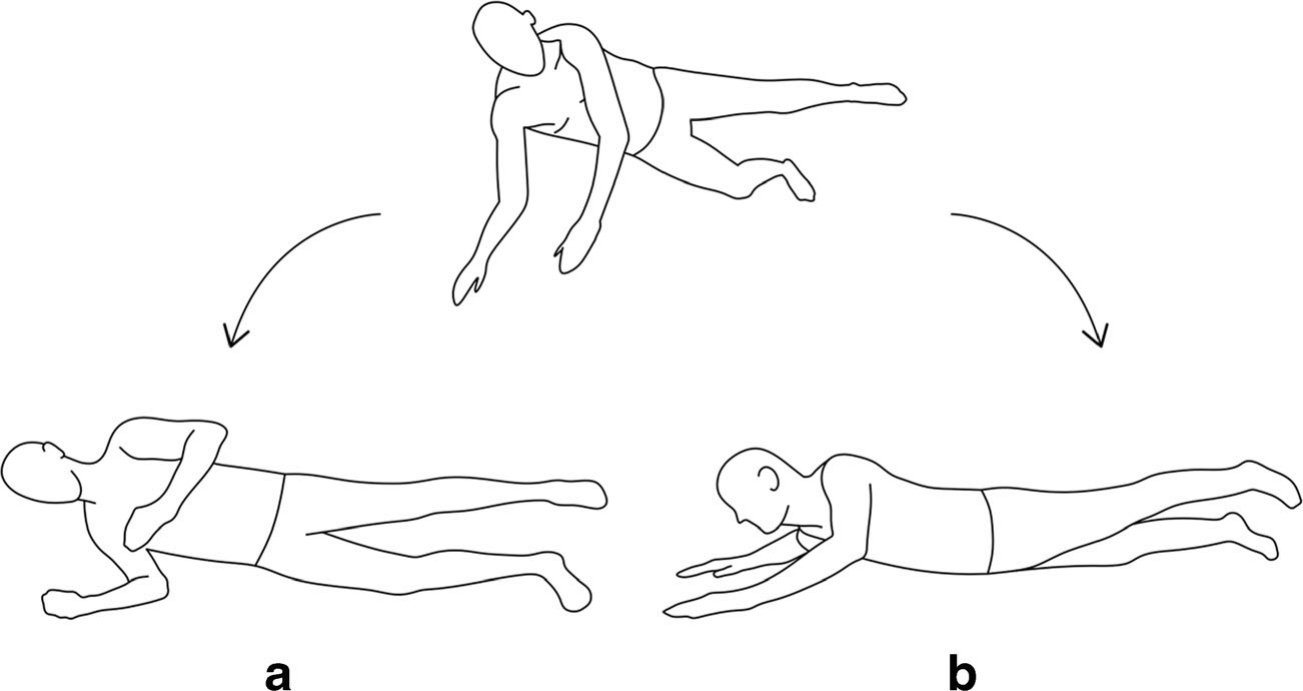
Drawings of a typical fall. The subject attempts to avoid to hit the floor with his face and uses the arms to attenuate the impact on the ground. Most falls are to the side (a), loading the posterosuperior rotator cuff or the front (b), loading the anterosuperior rotator cuff

Persons who slip or trip do not simply let themselves fall. By moving their arms, they try to find their balance again or reach for nearby support, such as a hand rail [80–82]. If the fall cannot be avoided, the arms are used as a protective measure to soften the impact on the ground and prevent injuries to the hips, shoulder, and head [83–85]. In the study by Lungren et al. [36], most falls were to the side and front. Accordingly, the arm impacting the ground may either be forced to the side [8, 77] or pushed away, resulting in an adduction and internal rotation or abduction and external rotation of the shoulder respectively (Fig. 2). In the first case, the supra- and infraspinatus tendons are most significantly stressed. In the second case, the subscapularis tendon and the rotator interval are subjected to heavy loads.

Several authors consider a fall as minor trauma [16–18, 24, 44]. This is not justified, as most fractures of the hip, the wrist, the olecranon, the humeral head, and the clavicle occur as a result of a simple fall. The forces occurring on impact may be considerable. Sabick et al. [86] simulated side falls from a kneeling position onto a force platform covered with foam. The peak impact force at the shoulder attained 3 times bodyweight (BW) when the subjects fell with the body tensed and 2.5 times BW when they attempted to break the fall by using an arm. Under the latter test condition, the peak force at the hand/arm was significantly greater than the force at either the hip or shoulder and averaged 3.78 BW. In a similar fall simulation using anthropomorphic test dummies, the peak impact force on the shoulder ranged from 3.2 to 10.1 times BW [87]. Naturally, the forces are expected to be much higher when falling from a standing or walking position [88, 89]. In vivo experiments with living people are not possible because of the risk of injury. Chiu and Robinovitch [89] therefore developed a computer model to predict the body’s impact response during forward falls from heights between 0 and 2 m and obtained a peak impact force on the hands of as much as 4.2 kN.

**Fig. 3:**
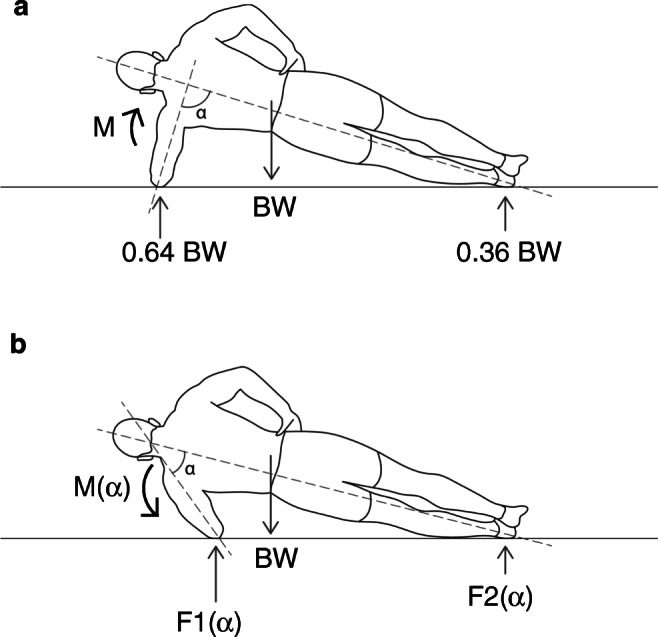
Illustration of the lateral plank exercise. When the arm is abducted 90°, the force under the elbow is about 0.64 body weight and directed to the shoulder. The moment in the shoulder is small and the body can be kept in balance. When the arm is brought against the body, the ground reaction force under the elbow F1(α) increases slightly, the lever arm and the moment M(α) in the shoulder to maintain balance, however, increase rapidly

**Fig. 4:**
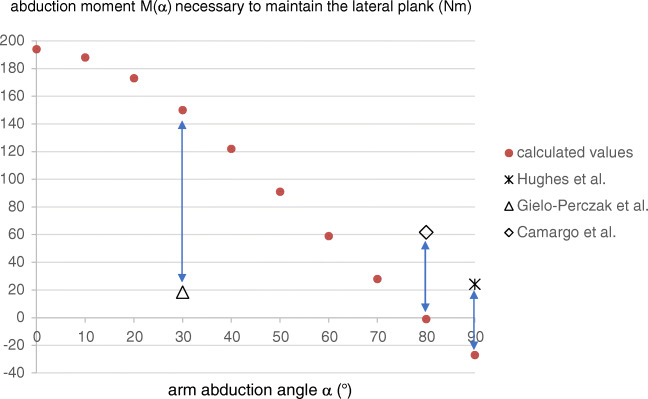
Graph representing the moment M(α) that the shoulder muscles must generate to keep a person weighing 84 kg in the lateral plank position as a function of the abduction angle (α) of the arm (red dots). The maximum moment that the shoulder muscles of healthy volunteers can generate is taken from previous studies and represented with separate marks. At an abduction angle of about 60° or less, the moment necessary to stabilize the body exceeds the moment that the shoulder muscles can generate and the arm is forced to the side, resulting in an eccentric loading of the rotator cuff and an impact of the shoulder on the ground

The forces occurring in the rotator cuff were not determined in these studies. The dynamic aspect of a fall, the numerous impact possibilities, and the energy absorption by the different tissues make the calculation of these forces very difficult. However, it is possible to determine the forces required to maintain the body in a lateral plank position. The lateral plank (or lateral abdominal bridge) is an isometric exercise to strengthen the oblique abdominal muscles and the entire core. The body is tensed and only the elbow, forearm, hand, and the lateral aspect of the foot are in contact with the ground. The supporting upper arm is almost perpendicular to the body axis (Fig. 3a). In this position, the ground force under the elbow is about 0.64 BW and is transmitted directly to the glenohumeral joint. The moment in the shoulder to maintain balance is small. The ground force F1(α) and the moment M(α) increase as the abduction angle of the arm becomes smaller (Fig. 3b and Fig. 4). They can be determined with trigonometry and with the equilibrium conditions. The maximum torque that the shoulder muscles can generate under isometric conditions has been determined by several researchers and varies between 18.5 and 61.7 Nm [90–92]. Accordingly, it is impossible for most people to keep the body in balance if the arm is spread 60° or less. When trying to slow down a fall, the peak forces at the elbow and the moment in the shoulder are even greater than under static conditions. When the moment exceeds the torque produced by the muscles, the arm is forced to the side and the posterosuperior rotator cuff tendon-muscle unit is lengthened while contracting. The sudden increase in stress may induce muscle damage and tendon lesions. That it is possible to tear a tendon with one’s own muscle strength can be observed with distal biceps tendon avulsions, quadriceps tendon lesions, and Achilles tendon ruptures. In all of these cases, the mechanism of injury is a sudden and high eccentric loading of the musculotendinous unit.

The mechanism of tendon rupture in vivo differs greatly from a monoaxial tensile test conducted in a laboratory. In vivo not all muscle and tendon fibres are equally stretched and stressed [93]. During eccentric contractions, the weakest elements will absorb most of the length change and may disrupt first [94]. The load is then distributed to the adjacent elements, which may in turn be overloaded and disrupt [8, 95]. The force needed to tear each fibre individually is much smaller than the force needed to tear all fibres simultaneously. Men who rip a phonebook in half with their bare hands take advantage of this feature (Video https://www.youtube.com/watch?v=k3yvxuMGwCg). Pre-existing alterations of the mechanical properties of the tendon tissue, such as altered gliding properties between tendon fibrils, may decrease its resistance [8, 95, 96].

The damage caused by a fall depends on many factors. These include, amongst others, the speed, height, weight, defense strategy, direction of impact, muscle strength, and tissue quality. It is conceivable that people who actively resist a fall are more likely to tear a tendon, while people who fall freely are more likely to suffer a humeral head fracture. The age of patients sustaining fall-related RCTs ranged from 15 to 89 [66] years. This highlights two important points: Firstly, the forces occurring during a fall can be so great that even a tendon of a young and healthy individual without degenerative changes can tear. Secondly, traumatic tears are not limited to young adults. Due to the greater risk of falling and the poorer tendon quality, the risk of traumatic tendon rupture is even higher in the elderly population [36].

Because most people fall one or more times during their lives, it is conceivable that many of the tendon tears classified as degenerative have been instigated by a fall and have been missed or trivialized at the time of the accident [38]. Zbojniewicz et al. [62] reviewed 205 MRI or MR arthrograms of children and adolescents at a large pediatric hospital and identified 25 RCTs. The majority were articular-side partial-thickness tears and treated non-surgically. As most RCTs do not heal spontaneously but enlarge over time, they may become symptomatic again years later when the patient no longer remembers the accident that originally weakened the tendon.

Our literature search does not claim to be complete. We have limited ourselves to the PubMed database and the terms “acute rotator cuff tear,” “traumatic rotator cuff tear,” and “fall AND rotator cuff tear.” Therefore, it is possible that numerous other studies reported fall-related tendon injuries that are not include in our review. Further cases and more consistent reporting of causes [97] would add to the evidence but would not change the fact that tendon ruptures can occur following a fall.

## Conclusion

Many clinical studies and biomechanical considerations confirm that a fall can cause an acute RCT and that fall-related tears are not restricted to young individuals. They can affect patients of any age. The forces and stresses occurring in the rotator cuff during an attempt to cushion a fall may locally exceed the tensile strength of the tendon fibres and cause a partial or full-thickness RCT.

## Data Availability

No supplementary material is submitted.
